# AULD: Large Scale Suspicious DNS Activities Detection via Unsupervised Learning in Advanced Persistent Threats

**DOI:** 10.3390/s19143180

**Published:** 2019-07-19

**Authors:** Guanghua Yan, Qiang Li, Dong Guo, Bing Li

**Affiliations:** 1 College of Computer Science and Technology, Jilin University, Changchun 130012, China; 2 Key Laboratory of Symbol Computation and Knowledge Engineering (Jilin University), Ministry of Education, Changchun 130012, China

**Keywords:** APT, DNS, unsupervised learning, behavior detection, sensor

## Abstract

In recent years, sensors in the Internet of things have been commonly used in Human’s life. APT (Advanced Persistent Threats) has caused serious damage to network security and the sensors play an important role in the attack process. For a long time, attackers infiltrate, attack, conceal, spread, and steal information of target groups through the compound use of various attacking means, while existing security measures based on single-time nodes cannot defend against such attacks. Attackers often exploit the sensors’ vulnerabilities to attack targets because the security level of the sensors is relatively low when compared with that of the host. We can find APT attacks by checking the suspicious domains generated at different APT attack stages, since every APT attack has to use DNS to communicate. Although this method works, two challenges still exist: (1) the detection method needs to check a large scale of log data; (2) the small number of attacking samples limits conventional supervised learning. This paper proposes an APT detection framework AULD (Advanced Persistent Threats Unsupervised Learning Detection) to detect suspicious domains in APT attacks by using unsupervised learning. We extract ten important features from the host, domain name, and time from a large number of DNS log data. Later, we get the suspicious cluster by performing unsupervised learning. We put all of the domains in the cluster into the list of malicious domains. We collected 1,584,225,274 DNS records from our university network. The experiments show that AULD detected all of the attacking samples and that AULD can effectively detect the suspicious domain names in APT attacks.

## 1. Introduction

With the development of the Internet of things, sensors in the Internet of things are commonly used in Human’s life, such as cameras in computers and temperature sensor, luminance sensor, and positioning system in mobile phones. However, because the security level of the sensors is relatively low when compared with that of hosts, Advanced Persistent Threats’ (APT’s) attackers often exploit their security vulnerabilities to achieve the purpose of invading hosts. In APT’s lateral movement stage, these sensor vulnerabilities are often exploited to install more backdoors to ensure long-term control over the target host. More and more sensors can connect to routers due to the appearance of Wireless Fidelity, which makes it possible for APT attacks to send information to servers through a sensor. This feature will be reflected in APT data communication stage. In the report [1], the attackers use the Internet of things to attack the target. They take advantage of weak passwords in cameras and routers to gain access to hosts and install malwares in them.

In recent years, various advanced persistent threats (APTs) have affected a large number of enterprises, organizations, and governments, such as OceanLotus [2], Harvest [3], and Hangover [4]. APT is a complex customized multi-stage attack. First, the attackers will carefully collect as much information of the potential attacking target as possible. Subsequently, they will launch customized attacks that can be diverse, such as spear phishing, watering hole, etc. [5]. Attackers lurk in the host for a long time to collect confidential information by using multiple malwares. Additionally, they use a variety of methods to send the stolen information to external servers. A series of APT attacks are closely linked and they are hidden in the targeted network. It is usually too late when the data leakage is detected.

Most APT attacks share two important behaviors: the various attack ways and long attack time. A variety of attack ways increase the infection possibility. The long attack time makes it possible to easily distribute the attack behaviors and lower the possibility of being detected. APT attacks can easily avoid the existing detection systems by mixing the malicious traffic in a large amount of normal traffic (such as firewall, intrusion detection system, etc.). The Mandiant takes four years and ten months from the preparation to the last data return [6].

The multi-staged APT attack usually includes four stages, preparation, attack, lateral movement, and data return [7]. In the preparation stage, attackers try to install malwares on users’ host by seducing them to visit the malicious websites. In the attack stage, attackers typically use such methods as waterhole attack, sending phishing e-mail, or taking a zero-day attack through the bugs of some softwares. In the lateral movement stage, the attackers obtain more information about the victim and lurk in victim hosts for a long time. In the data return stage, the attackers will send collected confidential information to external servers, during which DNS is usually to be used. Therefore, DNS plays a vital role in the whole APT attack process. Therefore, the detection of APT suspicious domains has become an important method for effectively detecting APT attacks. Currently, such suspicious domain detection methods are usually divided into three categories: graph-based method, machine learning, and credit rating. Graph-based methods mainly use the graphs to describe the relationships between the domains and extract features from the graphs to detect malicious domains [8]. Machine learning based methods mainly extract features that are based on the characteristics of APT in all aspects and use supervised learning or unsupervised learning to detect malicious domains [9,10]. For example, Gossip [11] proposes a method that is combined with natural language processing (NLP) and machine learning. NLP method is mainly used to detect malicious domains. Weina Niu [12] proposes an anomaly detection algorithm, Global Abnormal Forest (GAF), to detect malicious domains by domain’s scores. Although existing machine-learning-based malicious domain detection methods are effective, they still face two challenges: (1) the extracted features are based on one single point of time, without considering the time locality features of APT attacks; (2) the small number of the samples with ground truth (labeled) in APT attacks limits the use of supervised learning.

We find that the attack behaviors of APT are related to time to a certain extent, due to the long time span of APT. Therefore, we intend to focus on the extraction of time-related DNS features to perform the effective detection of APT. In particular, we extract three types of features that are based on host, domain and time. They are not only simple time point features, but also features presented in the prolonged cycle of APT attacks. However, the use of unsupervised learning is inefficient and low-accuracy in the absence of attack samples. We use an improved K-means algorithm based on density Canopy in order to improve the accuracy and stability of K-means algorithm and solve the problem of determining the most appropriate number K of clusters and best initial seeds [13,14]. Our method is motivated by the fact that literature [15] has shown that unsupervised learning can effectively detect APT behaviors.

In this paper, we propose a framework, APT Unsupervised Learning Detection (AULD) to detect APT attacks that are based on the features of DNS. It can detect the suspicious domains that are associated with APT attacks by unsupervised machine learning and better restore the whole process of APT attacks. Firstly, we preprocess the collected DNS request data. Afterwards, we extract ten features based on host, domain, and time. Subsequently, we use the unsupervised method to cluster the data and get the list of suspicious domains. Finally, we collect 70 days’ DNS request records, a total of 1,037,572,118, from our university campus network. We use the first 20 days’ data as the training data and the last 50 days’ data as the testing data. We use these data to evaluate our method.

The main contributions of this paper are as follows:

1. We propose a framework AULD to detect suspicious domains in APT activities, use the fully automatic unsupervised machine learning to analyze a large number of DNS log data, and obtain the list of suspicious domains.
2. We extract features based on host, domain, and time from the DNS log data according to attackers’ behaviors during the whole process of an APT attack, and then use them for clustering, in which the features that are based on time have not been mentioned by the existing work.
3. We verify the validity of our method and framework by conducting experiments with a large number of DNS log data collected from our university campus network.

## 2. Related Work

The existing researches on APT mainly focus on APT attack models and APT detection methods. Reviews [5] summarize the life cycle and the attacking principles of APT from its origin and development. They discuss the feasible defense system and detection method, but they do not provide the exact detection framework and method [16,17,18]. Most researches consistently divide an APT attack model into four stages: reconnaissance, compromise, lateral movement, and data return.

For detection methods, the existing work mainly uses methods, including machine learning, graph method, and credit rating. They often use one or two of the methods to detect APT attacks.

The main method of using machine learning for research is to extract APT-compliant feature vectors from DNS data. The researchers classify the domains according to the feature vectors and get the malicious domains related to APT activities. Yong Shi proposes a method for detecting malicious domains by using Extreme Learning Machine, which presents high precision and fast speed [19]. YI Nadji puts forward the system framework ghost&rae to manually detect APT attacks [9]. X Liang et al. proposed a Q-learning-based APT defense scheme that the storage defender can apply without being aware of the APT attack model or the subjectivity model of the attacker in the dynamic APT defense game is also proposed [20]. They extract the features from the latency period of APT for machine learning and obtain the external malicious domains that are associated with specific APT attacks. The framework only aims at the manual APT that was proposed by the author, so there are significant limitations on detection. C Huang designs a framework Gossip to detect malicious domains based on the analysis of discussions in technical mailing lists (particularly on security-related topics) by using natural language processing and machine learning techniques [11]. Gossip can find the malicious domains faster than network blacklist. Bohara extracts features from the graph his team proposed and uses K-means clustering method to effectively detect the behaviors in APT’s lateral movement stage [15], which also shows that unsupervised learning can detect APT behaviors well.

Researches based on the graph method mainly start with an undirected bipartite graph generated by host-domain and domain-IP. Researchers usually extract features from graphs and use machine learning or the determinate algorithms that they proposed for detecting the related APT activities. RSA proposes a new framework based on the belief propagation in graph theory to solve the problem of early detection of enterprise infection [21]. The framework can be used either with “seeds” of compromised hosts or malicious domains or without any seeds. They also set up a detector of command & control (C&C) to detect the communication between the host and C&C servers. The framework outputs the suspicious domains by the belief propagation algorithm. Websites that have never been accessed or been accessed for only as few times in internal network are the input data of the framework. B Rahbarinia puts forward the system Segugio that can effectively track the unknown domains maliciously controlled in internal network [10]. The system generates host–domain’s undirected bipartite graph by a large number of DNS data and then labels the nodes to extract features from the graph. The limitation of the method is that it depends on the accuracy of samples with ground-true. I Khalil proposes a determinate algorithm to detect unknown malicious domains, which assumes that the domains have a strong correlation with malicious domains [8]. They detect unknown malicious domains through a blacklist and determinate algorithm. The limitation of the method is that it can only be an auxiliary method to detect APT attacks.

The researches that are based on credit rating mainly put the extracted feature vectors into a credit rating system to score the domains that are to be detected and output suspicious domains or the ranking list of suspicious hosts. Mirco Marchetti puts forward a method to detect APT’s weak signals that were generated in the data return stage [22]. They extract the features in the header of IP packet and use credit rating to output a ranking list of suspicious internal hosts. It helps security officers to concentrate on the analysis of a small number of hosts, and it can detect the complete process of an APT attack faster. The limitation of this method is that the features that they extracted are few. They also put forward a new framework, AUSEPX, to detect hosts that may be infected in internal network [7], and the framework provides the ranking list of suspicious internal hosts by combining the calculation of internal host’s two indexes with the security intelligence. We put forward the framework APDD in another paper of our study that assists to detect hidden DNS suspicious behaviors in APT [23]. We collect a large number of DNS request data and extract all of the features conforming to the characteristics of APT attack. We use the method change vector analysis to analyze the similarity between the domain access record and the existing domain that was used by APT. We build the credit rating system to score the accesses of high similarity and then the system outputs a ranking list of suspicious domains’ access record. In terms of the combination of the above methods, A Bohara [15] uses the combination of graph method and machine learning and Guodong Zhao [24] uses the combination of credit rating and machine learning.

We summarize the features of the relevant work and present it in a tabular manner (see in Table 1).

The existing detection methods that are mentioned above are almost based on single-time nodes. For machine learning, most researchers use the supervised learning. We propose a framework AULD to assist to detect APT attacks in order to solve the problem that the attacking samples are few and to take the time locality feature into consideration. This framework adds the features that are based on time locality to the features in exiting work and it can help to detect suspicious domains in the long time span. It uses automatic unsupervised machine learning to cluster the domains that need to be detected and it is not limited by the samples with ground truth (labeled) and it improves the accuracy of the detection. It is a real-time detection that detects every day’s DNS data to find the suspicious domains of APT faster. It can let the defenders restore the whole APT attack process more quickly and minimize the loss.

## 3. The Framework of Auld

**Figure 1:**
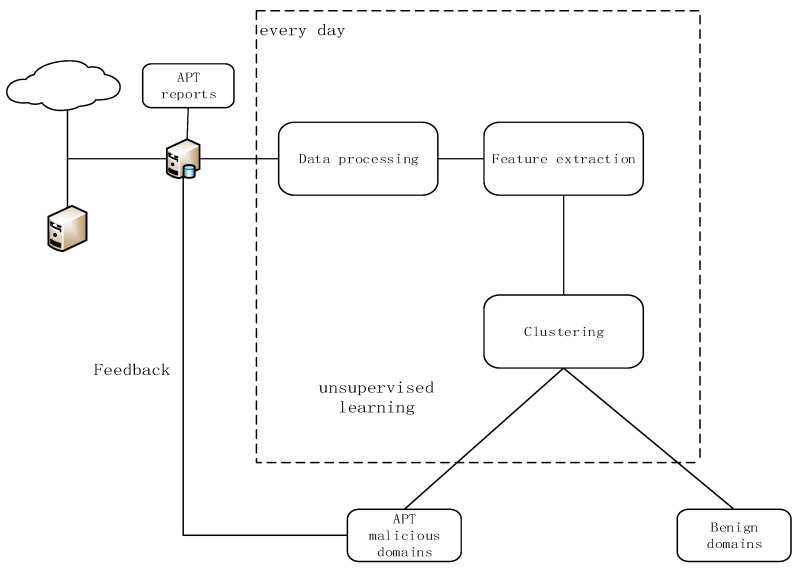
The flow diagram of Advanced Persistent Threats Unsupervised Learning Detection (AULD).

In this section, we introduce the detection framework AULD and describe each part of the framework in detail in the subsections. This framework is based on fully automated unsupervised machine learning, and it outputs the list of suspicious domains by analyzing the features of DNS log data. The test cycle of the framework is one day and the framework tests every day’s DNS data. It can report to the security officers timely when it finds suspicious domains, and it can help the defenders discover APT attacks faster. This framework consists of four parts: data collection, data preprocessing, feature extraction, and clustering. Figure 1 shows the flow diagram of our framework.

### 3.1. Data Collection

Through our university campus network, which is one of the largest campus networks in China, we collect a total of 1,037,572,118 pieces DNS request records (including wireless access) and use the data to test our framework. The IP field of the host is treated anonymously in order to protect individual privacy. The data used in this paper includes 70 days’ consecutive DNS log data from May 23, 2016 to July 31, 2016, which includes source port, the IP of the internal host the accessing domain, the accessing date, and other fields. We find that there is a clear time sequence among the APT attacks of the reports and that the reports give some related malicious domains as well as the corresponding accessing time through the analysis of recent APT attack reports [25,26]. According to the analysis of these reports, we generate six independent DNS simulation data without changing the domain access time in the literatures (label the simulation data from S1 to S6) and put them into the existing DNS request data [23].

### 3.2. Data Preprocessing

**Figure 2:**
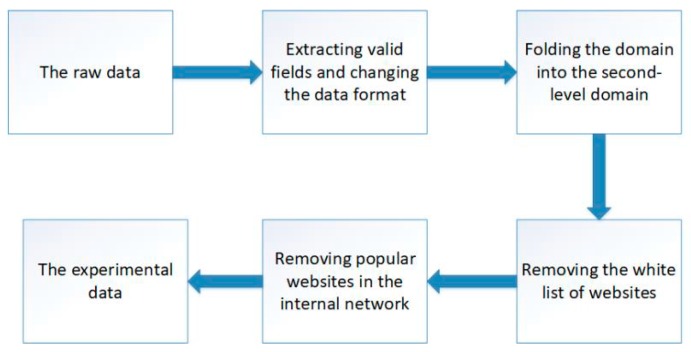
The process of data preprocessing.

Figure 2 shows the process of data preprocessing.

Data preprocessing consists of four parts: extracting valid fields and changing the data format, folding the domain into the second-level domain, removing the white list of websites, and removing the popular websites in the internal network. The preprocessed data is used for cluster analysis and to verify the validity of this method.

We introduce the four parts as follows:

Extracting valid fields and changing the data format: We extract three fields the IP of the internal host, the accessing domain, and the accessing time from DNS logs. Additionally, we standardize the accessing time to make processing more convenient.

Fold the domain into the second-level domain: We fold all of the domains into second-level domains, for example, the third-level domains www.zhidao.baidu.com and hexjlxm.zhidao.baidu.com are both folded into the second-level domain zhidao.baidu.com.

Removing the white list of websites: We remove the domains that are in the top one million websites of ALEXA, because attackers have to pay a great deal to launch attacks through these very popular websites.

Removing popular websites from internal networks: This stage of preprocessing is based on three rules: (1) Remove the request record of popular domain names. In the APT attack process, if the attacker wants to launch an attack by attacking a very popular website, the cost is too high, so it is considered that the very popular domain name’s request record is a good access behavior. (2) The number of accesses to the host has been >α times (for example, if the times that the host 11. 22. 33. 44 accessed apple.com on May 26, 2006 are more than α time, all records of the requests for the access to apple.com by 11.22.33.44 on 2016.5.26 will be deleted). An attacker will try to access an external C&C server or domain name as little as possible in order to avoid border detection. (3) Remove the record with the total access record less than β times in the detection time window. Obviously, hosts that are particularly inactive in long time windows are not helpful in the detection system.

### 3.3. Feature Extraction

We summarize three types of features based on host, domain, and time by considering the characteristics of the domains behavior in APT activities by analyzing a large number of APT reports [27] and according to the whole APT attack process. Among these features, feature 2, 9, and 10 all include APT’s characteristic of time locality and the feature 6 involves the characteristic of time interval. These all reflect that APT attackers lurk in targets for a long time and obtain information quickly in a short period of time. We select the relevant features according to this characteristic of time. Table 2 shows the feature set.

F1: The number of hosts that access the domains to be detected. APT has an obvious feature in the attack stage that the attackers only attack a single host or a small number of hosts because the attackers launch attacks purposely. The number of hosts infected by APT malicious domains is very small.

F2: Independent access. APT attackers try to avoid boundary detection and bypass the existing various security measures in internal networks. We set the access times of domains that are to be detected within time σ as the characteristic value of an independent access. The smaller the value is, the more suspicious the domain is.

F3: Access times of domain. In both the attack stage and the lateral movement stage, to avoid the detection, the attacker’s behavior is very covert and they rarely access external servers. Accordingly, the fewer times a domain is accessed, the more suspicious it is.

F4: Similarity of domains. Some attackers use the domains that are similar to the popular ones in the process of APT attacks, so the victims are easy to be seduced to access these domains when they do not act carefully. Hence, the higher the similarity between the domains to be tested and the popular websites, the more suspicious the domains are.

F5: Popularity of domains. The websites that internal hosts access frequently are credible. If the domains to be detected are never accessed or are accessed few times by internal hosts, they will be suspicious. Accordingly, the access time of domains to be detected can be the characteristic value of the popularity of domains.

F6: Automatic connection. Under normal conditions, the interval of the internal host’s accessing the same domain twice is not too short. In one host, if the interval between two domain accesses is less than 1 s, the domain is suspicious.

F7 and F8: Domain age and the expiration date of domain. We use these two features as the auxiliary features for improving the accuracy of clustering. F7 and F8 can be obtained by extracting the information of the domains’ WHOIS.

F9: Time point of access. In general, there are some regularities among the time points of domain accesses in internal hosts. It is suspicious that there are some domain accesses during abnormal time. We put the access times of the domains to be accessed in abnormal time points as the characteristic value. The higher the value is, the more suspicious the domain is.

F10: Time interval between two accesses of the domain to be detected. Attackers lurk in the host for a long period of time in the lateral movement stage. In this stage, the attackers conceal their own behaviors in the infected hosts and rarely access external servers. Hence, the longer the time interval of two domain accesses is, the more suspicious the domain is. We put the time interval between two accesses of the domain to be detected as the characteristic value.

We have found that using large feature sets does not improve accuracy and the use of some features adds noise. For example, the use of features of WHOIS and TTL. The characteristics of different websites will increase our false positive rate. The reason for this phenomenon is that the information provided by WHOIS and TTL in the report is not perfect in our simulation process. In the case where it is impossible to simulate the characteristics of malicious DNS behavior, we use the effective features that are reflected in the report for malicious behavior detection.

### 3.4. Complexity of Algorithm

The time complexity of the traditional Kmeans algorithm is O (nck), where n is the number of data objects, c is the number of iterations, and k is the number of classes. This paper introduces the Canopy clustering to generate k canopies, and each data object may belong to q (q ≤ k) canopies at the same time. The time complexity of the algorithm is O (ncq2k/p) when the number of clusters is p. It can be seen that the time complexity of the algorithm is significantly reduced compared to that of the traditional Kmeans.

### 3.5. Clustering Algorithm

In this paper, we use the mixed algorithm of Canopy and K-means due to the lack of attacking samples and the proof that unsupervised learning can effectively detect APT attack behaviors in existing work. The K-means algorithm is the most widely used algorithm among existing clustering algorithms. The K-means algorithm has the function of optimizing iteration and it can reduce the total time complexity of clustering for small samples. However, in K-means algorithm, the K value is set in advance, and the selection of K value is very difficult to estimate. The cost of the algorithm is huge when the data volume is quite large [28]. The Canopy algorithm is different from the traditional K-means algorithm. Its most obvious characteristic is that it does not need to give a K value in advance. The precision of Canopy is low, but there is a big advantage in the operational speed [29]. Accordingly, the Canopy algorithm is commonly used in clustering first to get the K value and then K-means algorithm is used to cluster. Therefore, the mixed algorithm of Canopy and K-means performs well in clustering.

When adjusting the training model, we perform parameter debugging for the distance centroid t1–t2 in Canopy algorithm, and evaluate the performance of the model in different parameter settings.

We introduce the clustering effect: ARI (Adjusted Rand Index) in order to express the performance level of the unsupervised training model we selected. We first introduce an element RI (Rand index) that constitutes ARI in order to explain ARI. We have the following definition:

RI = (a + b)/(C_2^(n_samples))(1)

In (1), C represents the actual category information, K represents the clustering result, a represents the logarithm of the same category of elements in both C and K, and b represents the logarithm of the elements of different categories in both C and K.

C_2^(n_samples) represents the logarithm that can be formed in the dataset and the range of RI is [0,1]. The larger the value, the more consistent the clustering result is with the real situation.

The larger the RI, the higher the accuracy of the clustering effect and the higher the purity within each class.

The adjusted rand index is proposed since the indicator should be close to zero in the case of random generation of clustering results, which has a higher degree of discrimination:

ARI = (RI-E[RI])/(max(RI)-E[RI])(2)

The range of ARI is [−1,1]. The larger the value, the more consistent the clustering result is with the real situation. From a broad perspective, ARI measures the degree of agreement between the two data distributions.

We set the value interval of t1–t2 to (0,10) and generate the following evaluation result graph that is based on the results.

**Figure 3:**
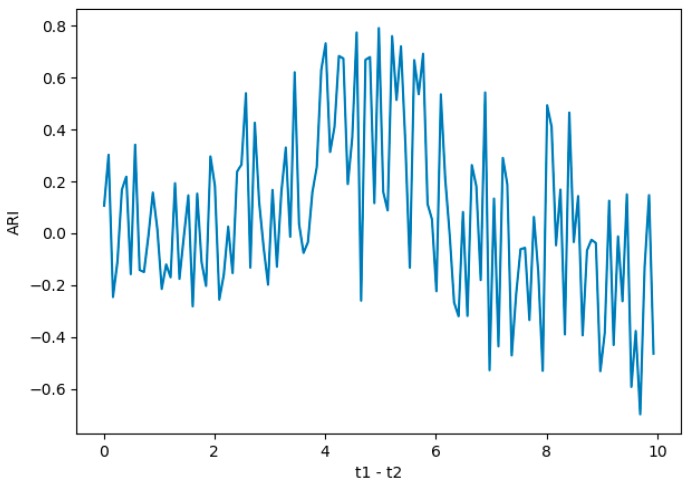
Parameter evaluation.

We conclude that the canopy model performs best when the parameter is set to 4.98(shows in Figure 3).

We also put a feedback system into the framework AULD. The framework outputs the report of APT malicious domains to reflect to the local APT repository. When we find the domains of the local APT repository again, we directly label them as suspicious domains. We remove the domains from the local APT repository until the security officers determine that the domains are irrelevant with APT activities.

## 4. Experiments

In this section, we introduce the details of the experiments’ process and results. We first introduce the situation of the data preprocessing and then analyze the results of the Canopy and K-means algorithm. Finally, we compare our experiments’ results with those of the other methods.

The purpose of the experiments can be concluded as the following three points:

1. explaining that the framework we proposed can be implemented in the environment of a real operating system;
2. verifying that the framework AULD proposed can effectively detect suspicious domains in APT activities; and,
3. comparing the advantages and disadvantages of Canopy + K-means algorithm with other algorithms.

### 4.1. Exprimental Environment

We deploy the framework AULD on a Win 10 system host with four cores eight threads, 16 gb memory, and 2t hard disk. It can be seen that the requirement of our framework’s hardware equipment is relatively low and the deployment is also very convenient. The clustering algorithms all run on WEKA (version 3.8.1).

### 4.2. Data Set Description

#### 4.2.1. Experiments’ Data Set

Table 3 shows the comparison between the initial data set and the data set that has been preprocessed and filtered by the features. Here we just show the average size of daily data. The first column represents the state of the data. The second column represents the number of domains and the third column represents the number of records. It can be seen from Table 3 that the data scale after data preprocessing and feature extraction is greatly reduced, which improves the efficiency of the subsequent clustering algorithm.

After the data preprocessing (in 3.2), we filter the logs that do not match our defined APT behavior. We will not detect single attacks or short-cycle attacks in the log, because the object that we are detecting is APT attacks, and the detected log must be consistent with the characteristics of APT attacks.

#### 4.2.2. Test Data Set

We constructed two data sets for experiments in order to test the feasibility and performance level of our designed framework: white dataset, black dataset.

White data set: For the construction of the white data set of this paper, we use the top one million whitelisted domain names of alex rankings for classification. We believe that so long as the access to the domain name in this whitelist is innocent.

Black data set: In the selection of black data, we refer to a large number of APT attack reports. Reading attack reports and related papers was undertaken to retrieve samples of available APT attacks. According to a report by Kaspersky Lab [30], we define a bunch of DNS traffic in Greece. This reflects the attacker’s transfer of the IP to the victim’s area via the groundwater server. We found a phenomenon in which an IP corresponds to two different domain names according to the report published by bae [25]. This report also points out that an attacker has a certain working time as a human being. This feature was also revealed by a report by Clearskysec [26]. We simulate this feature to some extent. Some malicious DNS traffic is mostly composed of multiple IPs parsed by a domain owner [31]. We also read other reports [32,33] to seek out the details of real APT attacks and simulate them.

**Figure 4:**
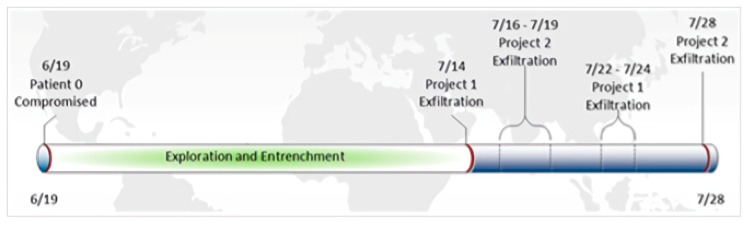
Data exfiltration timeline.

**Figure 5:**
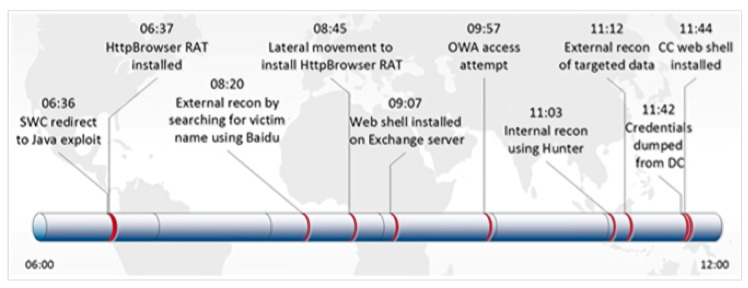
Timeline, in eastern-time, of TG-3390’s initial entry into a victim’s network.

**Figure 6:**
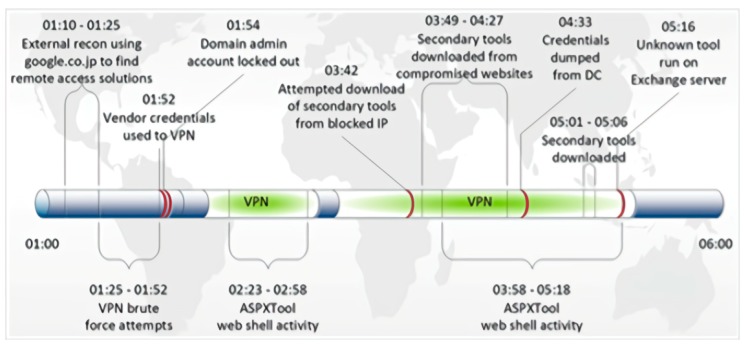
Timeline, in eastern-time, of TG-3390’s reentry into a compromised network.

We give an example of DNS log emulation in this paragraph. First, we read the report to find a report that is suitable for simulation. For example, in this report, the security team gave detailed attack cycles and details of the attack. The key attack behavior is expressed in the form of a timeline (see in Figure 4, Figure 5 and Figure 6). We simulated our DNS logs according to these timelines. At the same time, the report also provided us with a large number of malicious domain names. For example, in the following list of malware domain names, we can use the domain names in the list to simulate the domain names in the log, or we can simulate the domain names in the simulated log, according to the characteristics of them.

### 4.3. Data Set Description

We first construct the feature vectors of the domains that are to be detected according to 10 types of features that we proposed. Subsequently, we use the Canopy algorithm to cluster them. The framework we proposed aims to detect every day’s data. We just show the experiments’ results of the day of June 10, 2016 (the results contain the samples of attack simulation data flow S1, S4, S5). Table 4 shows the results of Canopy clustering. The first column represents the number of the clusters. The second column represents the number of instances and the third column represents the proportion of the instances in each cluster to the total.

Canopy algorithm chooses the method that is simple and needs fewer times of iteration to calculate the similarity of objects. It puts the similar objects into one subset and gets several subsets through a series of calculations. Table 3 shows that 14 subsets are obtained by the Canopy clustering. As the Canopy algorithm can only make a rough clustering, the effect is not satisfactory and the number of instances in each cluster has great difference. The simulation attack samples are not well clustered. Afterwards, we use the K-means algorithm to cluster the data set. We take the number of the subsets of Canopy clustering as the K value, which reduces the blindness of the selection of the K value to some extent. Table 5 shows the results and average characteristic values of K-means clustering. The first column represents the number of the clusters. The second column represents the number of instances in each cluster. The third to the tenth columns represent the average characteristic value of each feature in each cluster. F1 to F10 correspond to the 10 features in the third section. F1 represents the number of hosts. In the feature F2, we set the value of σ at 2 s and the unit of the parameter is time. In the feature F4, we get the value that is between 0 and 1 by the comparison between the domains and the white paper. The larger the value is, the higher the similarity between the domains and the white paper. F6 represents the time interval that one domain is accessed twice and the unit is second. F7 represents domain’s age, and we obtain the value by subtracting the domain’s registration time with the current time and the unit is day. F8 represents the expiration date of the domain. We obtain the value by subtracting the current time with the domain’s expiration time and the unit is day. In feature F9, we set the suspicious time at one point to six and the unit is time. The unit of the values in F3 and F5 is time and the unit of the value in F10 is day.

It can be seen from Table 5 that the average characteristic values of cluster 14 fit the characteristics of the APT attack mode well. In the cluster, the number of hosts accessing the domain, the average time of independent access and the number of domain accesses are all small. The similarity of the domains is slightly higher than the mean. There is not a particularly popular domain in internal networks. The time of automatic connection is less than the average. The domain age and validity period are relatively short. There are accessing behaviors in an abnormal time point. The interval of domain accesses is long. While considering all the features above, cluster 14 is the most suspicious. By observing the domains in cluster 14, we find that the simulated attack samples (S1–S6) are all in this set, which indicates that the hybrid Canopy + K-means algorithm can effectively detect the suspicious domains in APT activities.

We can see from the results of clustering that the average characteristic values of cluster 3, 11, 12 are similar to the values of cluster 14. The domains in cluster 3 are never accessed in internal networks, but the domains’ similarity is less and the average characteristic value of the independent accesses is too high. It does not conform to the characteristics of APT attacks. In cluster 11, we find that the domains are almost out of date and the time points of the accesses are normal. The similarity of domains is not high. In cluster 12, the time points of accesses are normal and the characteristic value of independent accesses is too high. To summarize, we can see that cluster 14 conforms to the characteristics of APT attacks most. We put all of the domains in cluster 14 into the list of malicious domains. It also shows that the effect of the clustering algorithm is good. We choose other clustering algorithms to deal with the data and, after comparing, we find that the effect of these algorithms, like DBSCAN and Density Peaks clustering (local density clustering), is general. They cannot effectively put the simulation attack samples into a cluster.

### 4.4. Comparison

**Figure 7:**
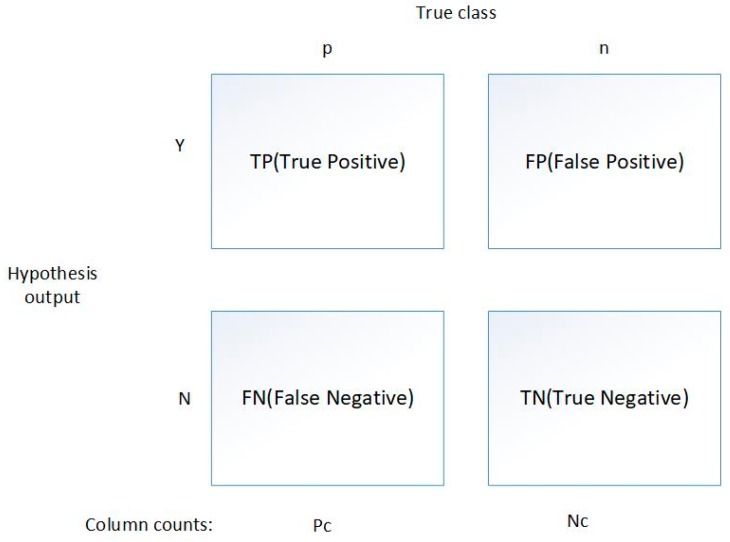
Confusion matrix.

(1) We used three standard evaluation parameters to evaluate the test framework we designed. The evaluation parameters include: TP, FP (shows in Figure 7), and Accuracy Rate. We introduce a confusion matrix to show the relationship between the false positive rate, false negative rate, malicious sample detection accuracy, and normal sample detection accuracy. Figure 7 determines these relationships. The false positive rate (FP-rate) indicates the proportion of innocent DNS behavior, which is determined by our detection framework to be malicious. The following formula 3 shows the relationship between these evaluation parameters.

Accuracy=(TP+TN)/(TP+TN+FP+FN)(3)

(2) We used the Canopy + k-means algorithm in this paper with several mainstream unsupervised learning and supervised learning to perform a ten-dimension verification on the dataset that we have built and labeled.

The supervised learning methods we choose are decision tree algorithm and logistic regression algorithm. Density-based algorithm DBSCAN and Density Peaks clustering (local density clustering) were the unsupervised learning methods we chose.

**Figure 8:**
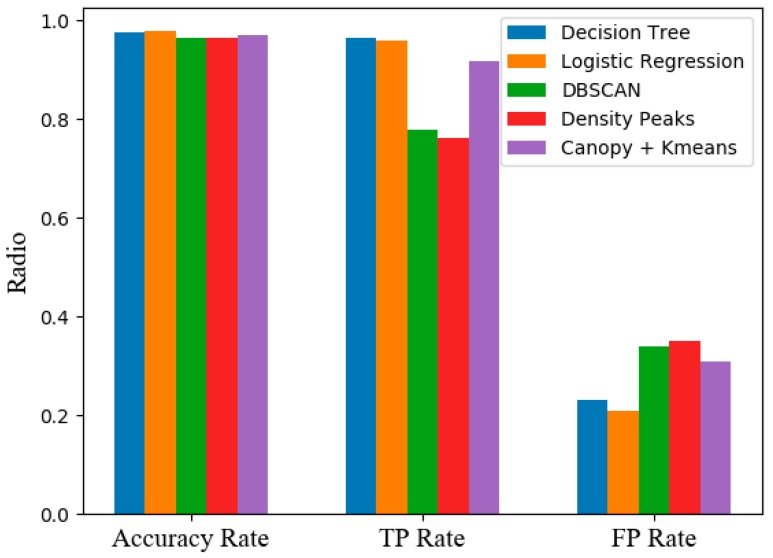
Experimental comparison (FP Rate is multiplied by ten for easier comparison).

We compare the FP-rate TP-rat and Accuracy of different models (see in Table 6). The experiments’ results are as follows:

We can see that the performance of the AULD architecture is lower than that of the supervised learning algorithm(shows in Figure 8). However, our algorithms perform better when compared to unsupervised learning algorithms. It can be concluded that our detection framework has higher detection performance in the absence of a label detection environment.

### 4.5. Algorithm Complexity Comparison

This section compares the time complexity of the algorithms used in the same kind of work. Table 7 shows the comparison structure in the following:

In Table 7, n represents the number of samples, C for a single sample, I for the number of iterations depends on the speed of convergence. M is the number of features, and D is the depth of the tree. The remaining elements can be viewed in 3.4. It can be seen that our algorithm is superior to the supervised learning algorithm in time complexity. It can be seen that the accuracy is superior to the unsupervised learning algorithm from the experimental results. 

### 4.6. Internal Problem

We filtered the high frequency access domain names located on the internal network. In the past attack cases, there are cases in which an attacker first sneaked into the internal network through the host managed by the internal network and attacked the target network segment through the internal network.

Accordingly, we re-added the filtered internal network data to our test data set and ran our test framework on this data set after completing all of the above experiments. The experiments’ results are shown in the following Table 8:

The test results show that there is a malicious domain name in the internal network, but the malicious behavior we detect is very rare because of the huge amount of white data. In this way, we can improve the model and data characterization in future research.

### 4.7. The Conclusion of Experiment

The experiments’ results show that the framework we proposed can effectively detect the suspicious domains in APT activities, and the security experts can further detect the domains in the list of suspicious domains to find the whole APT process. We compare the different clustering algorithms in the WEKA and choose the most popular K-means. We set the number of subsets of the clustering results of Canopy as the K value. The effect of the algorithm, combined with Canopy and K-means, is better than other clustering algorithms. According to the results, we can see that the framework AULD can effectively put the domains that correspond to the features of APT among the large number of domains into one category. Security experts can preferentially analyze these domains and interrupt APT activities as soon as possible to reduce the loss.

## 5. Conclusions

We propose a framework AULD, which is a method that assists in detecting suspicious domains in APT attacks since an APT attack has its time locality feature and unsupervised learning can effectively solve the problem that the samples with ground truth (labeled) are few. The list of suspicious domains output finally can help security officers to find and restore the entire APT attack process faster. AULD extracts features that are related to host, domain, and time in DNS request data, and it uses unsupervised machine learning to cluster to provide a list of suspicious domains. In this paper, we just propose an auxiliary method, and it cannot detect the complete process of an APT attack. In the future work, we will restore the entire APT attack process through the analysis of associated logs and the method that we proposed in this paper.

## Figures and Tables

**Table 1:** Feature summary.

Instances
F1: the fraction of known infected machines [10]
F2: the fraction of “unknown” machines [10]
F3: the total number of machines [10]
F4: total number of days in which d was actively queried [10] F5: the number of consecutive days ending with now in which domain was queried [10]
F6: the fraction of IPs that were associated to known malware domains [10]
F7: the fraction of such prefixes that match an IP that was pointed to by malware domains [10]
F8: URL features [11]
F9: Terms used in email subjects [11]
F10: Number of replies [11]
F11: Number of domains [11]
F12: Number of IP addresses [11]
F13: Number of attachments [11]
F14: Number of organizations [11]
F15: Number of known malware-detection services [11]
F16: Number of cryptographic digests [11]
F17: Participant features [11] F18: Text cleaning [11]
F19: Removing stop words [11]
F20: Stemming [11]
F21: Extracting contextual words [11]
F22: Number of distinct source IP addresses [11]
F23: Number of distinct IP addresses with the same domain [11]
F24: IP in the same country using the predefined IP addresses [11]
F25: Alexa ranking [12] F26: The length of domain [12] F27: The level of domain [12] F28: containing IP address [12] F29: Request frequency [12] F30: Reaction time [12] F31: repeating pattern [12] F32: Registration duration [12] F33: Active duration [12] F34: Update duration [12] F35: Number of DNS [12] F36: Length of domain [19]
F37: Number of consecutive characters [19]
F38: Entropy of domain [19]
F39: Number of IP addresses [19]
F40: Number of countries [19]
F41: Average TTL value [19]
F42: Standard deviation of TTL [19]
F43: Life time of domain [19]
F44: Active time of domain [19]

**Table 2:** Feature set.

Cluster	Instances
Host-based features	F1: The number of hosts that access the domains to be detected
	F2: Independent access
Domain-based features	F3: Access times of domain
	F4: Similarity of domains
	F5: Popularity of domains
	F6: Automatic connection
	F7: Domain age
	F8: The expiration date of domain
Time-based features	F9: Time point of access
	F10: Time interval between two accesses of the domain to be detected

**Table 3:** Average size of daily data.

Data	Domain	Records
raw data	17675	17009379
data preprocessing	9068	36821

**Table 4:** The results of canopy clustering.

Cluster	Instances	Percentage
1	6705	14%
2	20445	44%
3	12652	27%
4	3879	8%
5	427	1%
6	220	0%
7	201	0%
8	360	1%
9	916	2%
10	145	0%
11	194	0%
12	136	0%
13	91	0%
14	152	0%

**Table 5:** The average characteristic values and results of k-means clustering.

Cluster	Instances	F1	F2	F3	F4	F5	F6	F7	F8	F9	F10
1	7408	5	2.05	9	0.71	183	1	272	348	0	2
2	1128	5	21.02	8	0.72	178	6	2738	2503	0	2
3	5341	1	30.46	1	0.31	0	1	1102	524	0	30
4	846	13	6.12	19	0.72	431	6	2610	2694	0	1
5	3318	4	2.25	9	0.73	189	1	2688	947	0	2
6	3250	30	14.04	44	0.73	952	23	2403	984	1	1
7	2190	5	21.66	7	0.74	165	6	4264	890	0	2
8	5217	12	12.30	20	0.72	456	9	247	329	0	0
9	2295	4	22.09	6	0.81	176	5	38	47	0	1
10	2691	4	22.63	6	0.64	122	5	45	77	0	1
11	4603	1	4.05	1	0.51	0	0	980	493	0	29
12	1691	1	22.25	1	0.72	3	1	965	499	0	11
13	5534	5	22.48	7	0.72	163	6	1037	787	0	1
14	1011	1	5.86	2	0.70	20	1	760	478	2	11

**Table 6:** Comparison result.

	Accuracy Rate	TP Rate	FP Rate
Decision Tree	97.6%	96.5%	2.31%
Logistic Regression	97.8%	95.8%	2.1%
DBSCAN	96.4%	77.8%	3.4%
Density Peaks	96.5%	76.1%	3.5%
Canopy + Kmeans	96.9%	91.8%	3.1%

**Table 7:** Algorithm complexity comparison.

	Time Complexity
Decision Tree	O (n*M*D)
Logistic Regression	O (n*C*I)
DBSCAN	O (n^2)
Density Peaks	O (n^2*logn)
Canopy + Kmeans	O (n*c*q*2*k/p)

**Table 8:** Readd internal network data comparison.

	Input Data	Malicious Domain Names Dectected
Internal domain contained	11896	1689
Internal domain not contained	9068	1462
